# Phenotypic and Genotypic Analysis of Multidrug-Resistant* Mycobacterium tuberculosis* Isolates from Sudanese Patients

**DOI:** 10.1155/2017/8340746

**Published:** 2017-01-18

**Authors:** Solima M. A. Sabeel, Mohamed Ahmed Salih, Manasik Ali, Salah-Eldin EL-Zaki, Nadir Abuzeid, Zeinab Abubaker Mohammed Elgadi, Hisham N. Altayb, Asrar M. A. Elegail, Nuha Y. Ibrahim, Bahaeldin K. Elamin

**Affiliations:** ^1^Department of Microbiology, Faculty of Medical Laboratory Sciences, University of Khartoum, Khartoum, Sudan; ^2^Microbiology Department, Faculty of Medical Laboratory Sciences, Ibn Sina University, Khartoum, Sudan; ^3^Department of Bioinformatics, Africa City of Technology, Khartoum, Sudan; ^4^Sudan Academy of Sciences, Khartoum, Sudan; ^5^Department of Epidemiology, Tropical Medicine Research Institute, National Center for Research, Khartoum, Sudan; ^6^Department of Microbiology, Faculty of Medical Laboratory Sciences, Omdurman Islamic University, Khartoum, Sudan; ^7^Veterinary Research Institute, Khartoum, Sudan; ^8^Microbiology Department, College of Medical Laboratory Sciences, Sudan University of Science and Technology, Khartoum, Sudan; ^9^National Reference Laboratory-Tuberculosis (NRL-TB), National Public Health Laboratory, Khartoum, Sudan; ^10^Department of Microbiology and Parasitology, College of Medicine, University of Bisha, Bisha, Saudi Arabia

## Abstract

*Background.* Currently, mutations in* rpoB*,* KatG*, and* rrs* genes and* inhA* promoter were considered to be involved in conferring resistance to rifampicin, isoniazid, and streptomycin in* Mycobacterium tuberculosis* (MTB).* Objective.* The aims of this study were to detect the prevalence of first-line tuberculosis (TB) drug resistance among a group of previously treated and newly detected TB patients, to determine the association between prevalence of multidrug resistance (MDR) and demographic information (age and sex), to explain genes correlated with MDR* Mycobacterium tuberculosis*, and to characterize MTB via 16S ribosomal RNA (16S rRNA) analysis.* Methods.* A hundred MTB isolates from Sudanese pulmonary TB patients were included in the study. The proportional method of drug susceptibility test was carried out on Löwenstein-Jensen media. Multiplex PCR of* rpoB* and* KatG* genes and* inhA *promoter was conducted; then* rrs* genes were amplified by conventional PCR and were sequenced. The sequences of the PCR product were compared with known* rrs* gene sequences in the GenBank database by multiple sequence alignment tools.* Result.* The prevalence of MDR was 14.7% among old cases and 5.3% among newly diagnosed cases.* Conclusion.* Mutations in* rrs* could be considered as a diagnostic marker.

## 1. Introduction

Monitoring of tuberculosis (TB) caused by drug-resistant* Mycobacterium tuberculosis* (MTB) has become one of the major problems throughout the world [1]. However, the detection of drug-resistant phenotypes of MTB takes at least 3–6 weeks by direct and indirect methods, respectively. Thus, treatment was prescribed empirically. Patients who fail to respond to drugs remain infectious [2]. They may be a source of transmission of infections [3].

Sudan is surrounded by high burden countries [4] and it harbors a high TB incidence among the East Mediterranean countries. The prevalence of TB infection in Sudan is probably variable in different regions [5]. The country has been severely affected by war, famine, and flood in recent decades and has a large population of internally displaced persons, which is considered risk factor of spreading TB. Cases among men exceeded those found in women by a ratio of 2 : 1 [6].

Multidrug-resistant TB (MDR-TB) is defined as MTB that is resistant to first-line anti-TB drugs such as rifampicin (RIF) and isoniazid (INH) [7]. RIF is a broad spectrum antimicrobial agent, which remains the most effective drug against* M. tuberculosis*. Resistance of rifampicin occurs via mutation in* rpoB* gene that encodes the *β*-subunit of RNA polymerase [8]. INH has the most powerful bactericidal activity against TB and has good tolerance and low price [7]. It is a prodrug, requiring activation through oxidation by a mycobacterial catalase-peroxidase enzyme encoded by* katG* gene [9]. Activated isoniazid interferes with the biosynthesis of essential mycolic acids through inhibition of nicotinamide adenine dinucleotide hydrogen (NADH) dependent enoyl-acyl carrier protein reductase, which is encoded by* inhA* promoter. Alterations in* katG* gene and* inhA* promoter were strongly associated with isoniazid resistance [10, 11]. Streptomycin (SM) was the foremost antibiotic successfully used against TB. Resistance to SM emerged as a result of monotherapy administration [12]. SM is an aminocyclitol glycoside that acts against actively growing bacilli by inhibiting the initiation of translation in protein synthesis at the level of the 30S subunit of the ribosome, specifically at the ribosomal protein S12 and the 16S rRNA coded by* rpsL* and* rrs* genes, respectively [13, 14]. Consequently, mutations in* rpsL* and* rrs* are the major mechanisms of resistance [10]. The aims of the current study were to detect the prevalence of monoresistance and multidrug-resistant* Mycobacterium tuberculosis* (MDR), to determine the association between prevalence of MDR and demographic information (age and sex), to explain the molecular characterization of* M. tuberculosis* through 16S rRNA, and to illustrate the frequency of* rrs *mutations among streptomycin-resistant* M. tuberculosis* isolates.

## 2. Materials and Methods

This study was approved by the Ethics Committee of the Africa City of Technology and the University of Khartoum, Sudan.

### 2.1. *Mycobacterium tuberculosis* Growth Properties

A hundred sputum samples were collected from pulmonary TB patients as a cross-sectional descriptive study conducted at the National Reference Laboratory-Tuberculosis (NRL-TB), Khartoum, Sudan. Samples were processed under biosafety cabinet level II via adding twice the volume of 4% NaOH to sputum in 50 mL centrifuge tube for decontamination and homogenization. Samples were neutralized by buffer, followed by centrifugation at 3000 RCF (Relative Centrifuge Force) for 20 min. Inoculation was adjusted by pipetting 3–5 drops of deposit in three Löwenstein-Jensen media (two containing glycerol and one containing pyruvate) which are egg based media. Then, cultured media were incubated at 37°C in slant position. Subsequently, weekly observation was made to check the growth of bacteria [15–17].

### 2.2. Drugs Susceptibility Test

Drugs susceptibility test was done through the conventional proportional method on Löwenstein-Jensen (LJ) media containing drugs according to CDC standard procedures [16, 18]. The grown colonies were picked up from the media and emulsified in a thick wall glass tube, containing DW + glass beads, by shaking the tubes well; furthermore, turbidity was adjusted with McFarland standard (number 0.5). The diluted suspension 10^−4^ was cultured on LJ drug-free media as a control and drug containing 0.2 *μ*g/mL isoniazid (INH), 40.0 *μ*g/mL rifampicin (RIF), 4.0 *μ*g/mL streptomycin (SM), and 2.0 *μ*g/mL ethambutol (EMB). The cultured media were incubated at 37°C and were observed after 4 weeks. If there are no colonies or if the ratio between the number of colonies in the media containing drug and the number of colonies in drug-free media is less than 1%, it will be considered sensitive, while if the ratio between the number of colonies in media including drug and the number of colonies in drug-free media is more than 1%, it will be interpreted as resistant to all four drugs. Species identification of* Mycobacterium* was carried out via testing the ability to grow on* p*-nitrobenzoic acid (PNB). All isolates were tested twice in media to confirm the accuracy of the results [19, 20].

### 2.3. Guanidine Chloride DNA Extraction Method

All scraped colonies were washed with phosphate buffer saline (PBS), followed by the addition of 2 mL lysis buffer, 5 *μ*L proteinase K, 1 mL guanidine chloride, and 300 *μ*L ammonium acetate. Suspensions were incubated overnight at 37°C; on the next day, 2 mL of chilled chloroform was added. After centrifugation, the clear upper layer was collected in a new tube and cold absolute ethanol was added to enhance precipitation of DNA. The pellet was washed with 70% ethanol and then decanted by 70% ethanol and allowed to dry. The pellet was then resuspended with nuclease-free water and qualified using NanoDrop ND-1000 spectrophotometer (NanoDrop Technologies, USA). The DNA samples were stored at −20°C until used for conventional PCR [21, 22].

### 2.4. GenoLyse Extraction Method

DNA extraction was performed as recommended by the manufacturer (Hain Lifescience). Firstly, collected bacterial colonies were heated for 20 minutes at 100°C in a water bath and then suspended in 100 *μ*L of kit lysis buffer, followed by incubation at 95°C for 5 min; finally, 100 *μ*L of neutralization buffer was added. The mixture was spun at full speed in a tabletop centrifuge with an aerosol tight rotor and then stored at −20°C and then used for multiplex PCR in Line Probe Assay [23].

### 2.5. Conventional PCR

Fifteen genomic DNA were used as templates for PCR amplification of complete* rrs* gene (16S rRNA). The two primers used were forward primer, namely, 27F (5′-AGAGTTTGATCCTGGCTCAG-3′), and reverse primer, namely, 1495R (5′-CTACGGCTACCTTGTTACGA-3′). The 25 *μ*L reaction mixture contained 1 *μ*L DNA, 1x reaction buffer (10x) with 3 mM MgCl_2_, 2.5 U i-Taq™ DNA polymerase (5 U/*μ*L), 2.5 mM dNTPs, 1 *μ*L of 10 pmol of each primer, and 1x of gel loading buffer, followed by completing the volume to 25 *μ*L by DW. PCR amplifying procedure was as follows: 5 min at 94°C, 30 cycles of 1 min at 94°C, 1 min at 58°C, 2 min at 72°C, and then 10 min at 72°C, which was performed on a Bio-Rad (DNA engine/Dyad Peltier) automatic thermal cycler. Duplicate PCR of every sample were carried out for more confirmation. The products of amplification were checked through running on 0.6% agarose gel electrophoresis [24, 25].

### 2.6. Multiplex PCR

Hybridization and detection were performed using the hybridization kits. Seventy-five isolated DNA were used by taking 5 *μ*L of each DNA and mixing it with 10 *μ*L Amplification Mix A and 35 *μ*L Amplification Mix B containing biotinylated primers. The PCR amplifying procedure was as follows: 15 min at 95°C, 10 cycles of 30 seconds at 95°C, 20 cycles of 40 seconds at 50°C, and 8 min at 70°C, which was performed on a Hain Lifescience thermal cycler. The amplification product was visualized through reverse hybridization probes complementary to amplified nucleic acids on membrane strips. [26].

### 2.7. Sequencing of 16S rRNA

Isolates were packaged according to the International Air Transport Association guidelines and shipped with authorized permission to Macrogen Company (Seoul, South Korea). Purification and standard forward sequencing of 16S rRNA were done by ABI Genetic Analyser (Applied Biosystems).

### 2.8. Statistical and Bioinformatic Analysis

The result was analysed statistically using IBM SPSS Statistics version 21 (Statistical Package for the Social Sciences) which is a software package for statistical analysis; the chi-square test was used to check the statistical significance [27]. The chromatogram sequences were visualized through Finch TV program version 1.4.0 [28]. The nucleotides sequences of the* rrs* gene were searched for sequences similarity using nucleotide BLAST [29]. Highly similar sequences have accession numbers HM007576, KF796661, JX303293, and GU142936 and sequences of the reference* M. tuberculosis* H37Rv strain [X55588.1] were retrieved from NCBI (https://www.ncbi.nlm.nih.gov/) and subjected to multiple sequence alignment using BioEdit software version 7.2.5 [30]. Newick format was withdrawn from ClustalW (http://www.ebi.ac.uk/Tools/msa/clustalw2) [31] to create a phylogenetic tree in Phylogeny.fr software [32].

### 2.9. Nucleotide Sequence Accession Numbers

The nucleotide sequences of the* rrs* genes containing novel mutations were deposited in the GenBank database (National Center for Biotechnology Information; https://www.ncbi.nlm.nih.gov/) under the following accession numbers: KU372152, KU372153, KU372154, KU372155, KU372156, and KU372157.

## 3. Results

Among 100 sputum samples, twenty of them revealed no growth, two were contaminated, one was lysed, and two had grown in PNB indicating* Mycobacterium* other than tuberculosis (MOTT). The growth rate on LJ media is as follows: 39% (29/75) showed +3 (200–500 colonies), 33% (25/75) showed +2 (100–200 colonies), 26.7% (20/75) showed +1 (10–99 colonies), and 1.3% (1/75) showed 4 colonies. Most of the MDR growth appeared as +1 and probably had grown at 2–4 weeks (*P* value 0.426), showing a statistically insignificant association between growth rate and MDR. The prevalence of MDR was 20% (15/75) and 14.7% (11/15) among previously treated and old cases and 5.3% (4/15) among newly diagnosed cases (*P* value 0.003), which means drug resistance was associated with previous treatment. 73.3% of MDR cases were males, while 26.7% were females (*P* value 0.536). Sixty percent (9/15) of MDR cases were found in the age group ≤ 30 years (*P* value 0.652) (Table 1). Among seventy-five* Mycobacterium tuberculosis* isolates, 56% (42/75) were streptomycin-resistant, 29% (22/75) were rifampicin-resistant, 20% (15/75) were ethambutol-resistant, and 28% (21/75) were isoniazid-resistant. 20% (15/75) were resistant to RIF and INH. 16% (12/75) were resistant to all four drugs. The run progression of the final separation of PCR products was visualized under UV transilluminator documentation machine. Most of them fluoresced sharply, but two of them fluoresced faintly and were excluded. The most common mutation detected by LPA in the* rpoB* gene was S531L (80%, 12/15), followed by H526D; in* KatG* gene, the most common mutation was S315T (67%, 10/15) and in* InhA* promoter it was C15T (33.3%, 7/21).

Sixty-two percent (8/13 isolates) of streptomycin-resistant isolates revealed mutations in* rrs* gene which were identified in seven groups: four isolates (31%) have G → A transition at nucleoside position 892; three isolates (23%) have C → A transversion at nucleoside position 222; two isolates (15%) have A → G transition at nucleoside position 904; one isolate (8%) has G → T transversion at nucleoside position 855; one isolate (8%) has A → G transition at nucleoside position 906; one isolate (8%) has T → A transversion at nucleoside position 1238; and two isolates have G → A/G → C at nucleoside position 1400, which are more explained in Table 2.

### 3.1. Phylogenetic Tree

**Figure 1:**
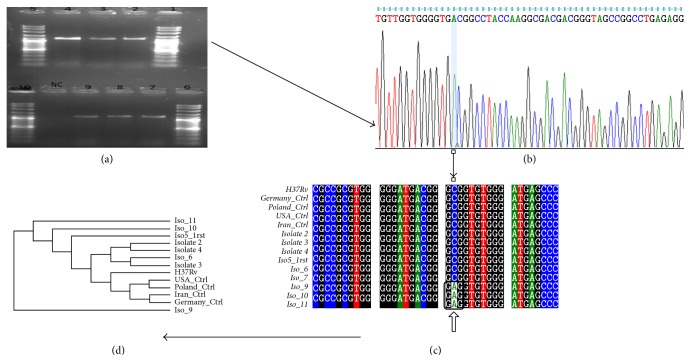
(a) Gel electrophoresis of PCR product. Lanes 1 and 5: DNA marker 1000+. Lanes 2, 3, and 4: amplified DNA products, 1500 bp. (b) Chromatogram sequence of 16S rRNA visualized via Finch TV software. (c) Alignment determining novel mutation at position 222, transversion of C to A in isolates 9, 10, and 11 via BioEdit software. (d) Phylogenetic tree using Phylogeny.fr software.

A phylogenetic tree represents the relationships among a set of* Mycobacterium tuberculosis*. The tree is classified into two branches. All isolates have a common ancestor except for isolate-9 which was outgroup. Isolate-2 and isolate-4 were sister groups as shown in Figure 1.

## 4. Discussions

The study found novel transversion mutation of C → A at position 222. To our knowledge, the present study is the first study that demonstrated 16S rRNA analysis among multidrug-resistant* M. tuberculosis* isolates from Sudan, and thus the prevalence of MDR was 20% (15/75) and 14.7% (11/15) among old cases and 5.3% (4/15) among newly diagnosed cases, which was lower than France's study with a prevalence of 23/323 (7.1%) in newly diagnosed patients and 33/105 (31.4%) in re-treated patients [33]; this difference could be due to the geographical variation. In the present study, 73.3% of MDR cases were males, while 26.7% were females, which agreed with Raizada et al.'s study where MDR-TB was predominant in males (72%, 230/320) [34] contrary to Melzer et al.'s study where 56.7% in males and 43.3% in females [35] may be due to the large numbers of males when compared with females included in this study. Most MDR cases were found in the age group ≤ 30 years (60%), corresponding with Melzer et al.'s study [35]. The genotype MTBDR*plus* test identified most frequent mutations involved in resistance to RIF and INH as follows: in* rpoB* gene, S531L was 54% (12/22); this is similar to the findings of Barnard et al. [36]. The final result of mutation frequency in* rrs* gene is 62% which is approximately similar to Asho's study conducted in Pakistan that detected 35/50 (70%) strains [37]. Also, additional studies in China, Japan, and Latvia have reported the highest frequencies, 85.7%, 77.8%, and 85%, respectively [38–40], whereas, in North India, no mutation had been detected in streptomycin-resistant isolates [41]. Regarding mutation in the 912 region, 23% of the isolates have been revealed. A → G transition at position 904 corresponds to studies in Germany [42]. On the other hand, a study in Barcelona did not detect mutation in the rrs912 region [43]. The mutation at loop 530 of the* rrs* coding region had not been identified, which agreed with New York's study [44] and conflicted with Poland's study [45]. Novel mutation could be used as a diagnostic marker that represents a tool for rapid monitoring of streptomycin resistance and could be of value to the clinician.

Small sample size was one of the drawbacks that limited our study; therefore, a large sample size in further studies could be useful for the determination of other biomarkers that assist the diagnosis of streptomycin-resistant MTB.

## 5. Conclusion

Analysis of 16S rRNA sequences is considered the golden standard method for the identification and assessment of phylogenetic relationships among bacterial isolates.

## Figures and Tables

**Table 1:** Percentage of MDR in different age groups.

Age group	MDR %	Account (number)
≤30	60%	9
31–40	13.3%	2
41–50	13.3%	2
51–60	13.3%	2
>60	0%	0

**Table 2:** Explaining the position and frequency of polymorphism with resistance pattern of isolates.

Mutation position	Polymorphism	Frequency (%)	Resistance pattern			
			RIF	INH	STM	EMB
222	C → A	23%	*R*	*R*	*R*	*S*
			*S*	*S*	*R*	*S*
			*R*	*S*	*R*	*S*

885	G → T	8%	*S*	*S*	*R*	*S*

892	G → A	31%	*S*	*R*	*R*	*S*
			*S*	*S*	*R*	*S*
			*R*	*R*	*R*	*S*
			*R*	*R*	*R*	*R*

904	A → G	15%	S	R	R	S
			R	R	R	R

906	A → G	8%	S	R	R	S
	T → A	8%	R	S	R	S

1400	G → A	8%	R	R	R	R
	G → C	8%	S	S	R	S

R: resistance; S: sensitive; RIF: rifampicin; INH: isoniazid; STM: streptomycin; EMB: ethambutol.
